# Integrated Fault Diagnosis Algorithm for Motor Sensors of In-Wheel Independent Drive Electric Vehicles

**DOI:** 10.3390/s16122106

**Published:** 2016-12-12

**Authors:** Namju Jeon, Hyeongcheol Lee

**Affiliations:** 1 Department of Electrical Engineering, Hanyang University, Seoul 133-791, Korea; sohorain@hanyang.ac.kr; 2 Department of Electrical and Biomedical Engineering, Hanyang University, Seoul 133-791, Korea

**Keywords:** high-level fault diagnosis, low-level fault diagnosis, integrated fault diagnosis, residual, fault table, fault-detection flag

## Abstract

An integrated fault-diagnosis algorithm for a motor sensor of in-wheel independent drive electric vehicles is presented. This paper proposes a method that integrates the high- and low-level fault diagnoses to improve the robustness and performance of the system. For the high-level fault diagnosis of vehicle dynamics, a planar two-track non-linear model is first selected, and the longitudinal and lateral forces are calculated. To ensure redundancy of the system, correlation between the sensor and residual in the vehicle dynamics is analyzed to detect and separate the fault of the drive motor system of each wheel. To diagnose the motor system for low-level faults, the state equation of an interior permanent magnet synchronous motor is developed, and a parity equation is used to diagnose the fault of the electric current and position sensors. The validity of the high-level fault-diagnosis algorithm is verified using Carsim and Matlab/Simulink co-simulation. The low-level fault diagnosis is verified through Matlab/Simulink simulation and experiments. Finally, according to the residuals of the high- and low-level fault diagnoses, fault-detection flags are defined. On the basis of this information, an integrated fault-diagnosis strategy is proposed.

## 1. Introduction

Following the recent increase in international oil prices and environmental issues, studies on the introduction of environment-friendly vehicles such as fuel-cell and electric vehicles have increasingly become popular. Among these vehicles, the in-wheel independent drive electric vehicles contain motors installed inside the wheels, which feature enhanced system efficiency and driving performance [1,2,3,4].

However, because the motor is installed inside the wheel, it is vulnerable to numerous faults due to exposure to harsh conditions such as physical impact, rapid temperature change, and variation in humidity. Therefore, to improve vehicle safety, fault diagnosis of the drive motor has become increasingly important.

The fault-detection methods can be mainly classified into hardware and analytic redundancies. Hardware redundancy uses an identical sensor or actuator to easily cope with the fault, which is an advantage over analytic redundancy. However, additional costs and installation space are required. In automobiles, analytic redundancy, which considers the information and system dynamics with respect to the system, is often adopted instead of hardware redundancy. Furthermore, in the present study, analytic redundancy is used to perform fault diagnosis without additional sensors [5].

Numerous studies regarding fault diagnosis of the drive motor are currently being conducted. In some studies, the position sensor value of the motor is estimated using a sensor-less method and is compared with the actual sensor value for fault diagnosis and fault tolerance [6,7]. The current sensor value of the motor is estimated for fault diagnosis using the proportional–integral [8] and the sliding-mode [9,10] observers. In other studies, the parity equation was used to diagnose the faults in electric current and position sensors [11,12]. In other studies, optimal design, including the number of motor slots and coil arrangement, was conducted to enhance reliability and fault tolerance [13,14]. In some works, the switch fault of a motor system and the detection and tolerance of an electric-sensor fault as well as that of the resolver/encoder through additional configuration were investigated [15,16,17]. Another research used frequency analysis of the electric current to diagnose a fault while the vehicle was not running [18]. Similarly, the drive motor fault diagnosis was mainly conducted at a low level of the system. However, when low-level fault diagnosis is conducted using a single model, a false alarm could occur because external noise and system uncertainty can be unnecessarily detected [19,20]. Therefore, to increase robustness, an additional high-level fault diagnosis is required [21].

Numerous fault diagnosis control strategies for vehicles have been suggested in ref. [22,23,24,25]. However, most of these research works dealt with conventional vehicles, but not in-wheel independent drive electric vehicles.

Several studies regarding fault diagnosis of the in-wheel independent drive electric vehicles are being conducted using a planar two-track non-linear model [26,27,28,29,30,31]. This planar two-track non-linear model, which can realize the longitudinal and lateral forces in each wheel, is widely used considering the characteristics of the in-wheel system in that each wheel drives independently. In one study, an actuator-grouping sliding mode controller was used for fault tolerant control [26], but it is assumed that the location of the specific faulty wheel is known. In other works the references [27,28,29,30,31] assumed that the vehicle dynamics sensors, such as the yaw-rate, longitudinal and lateral acceleration, and wheel-speed sensors, are at a non-fault state. However, if dynamic sensors fail, the fault diagnosis algorithm proposed in previous research [27,28,29,30,31] cannot diagnose the motor sensor fault.

Accordingly, in the present study, the diagnosis system integrates both the high-level fault diagnosis of the vehicle dynamics and low-level fault diagnosis of the motor system by considering the vehicle dynamic sensor faults to propose a method that increases the robustness and stability of the system.

For the high-level fault diagnosis of the vehicle dynamics, a planar two-track non-linear model is selected, and using a non-linear tire model, the longitudinal and lateral forces are calculated. By utilizing the motor drive torque and wheel velocity, the wheel dynamics is configured in determining the residual. To increase the redundancy of the system, the correlation between the sensor and residual in the vehicle dynamics is analyzed to detect and separate the fault in the drive motor system in each wheel and the vehicle dynamic sensors such as the yaw-rate, longitudinal and lateral acceleration, and wheel-speed sensors. The low-level fault diagnosis of the motor system is performed by configuring the state equation of an interior permanent magnet synchronous motor (IPMSM) to detect and separate the faults of the electric current and position sensors. Finally, by preparing a dual fault monitoring structure combined with the high- and low-level fault diagnoses, the robustness and stability of the diagnosis are reinforced while allowing the system to perform a specified classification of the fault.

This paper is divided into the following sections: In Section 2, the high-level fault diagnosis of vehicle dynamics is proposed, and the validity of the algorithm is verified by Carsim and Matlab/Simulink co-simulation. In Section 3, the low-level fault-diagnosis method of a motor system is proposed, and the validity of the algorithm is verified through Matlab/Simulink simulation and experiments. In Section 4, the high- and low-level fault-diagnosis systems are integrated to propose detailed detection and diagnosis methods.

## 2. High-Level Fault Diagnosis

For the high-level fault diagnosis of the vehicle dynamics, a planar two-track non-linear model, which can realize the longitudinal and lateral forces in each wheel, is used considering the characteristics of the in-wheel system that each wheel drives independently. By analyzing the influence of the drive motor fault on the entire vehicle, a correlation between each sensor and the realized residual is derived.

### 2.1. Planar Two-Track Model

**Figure 1:**
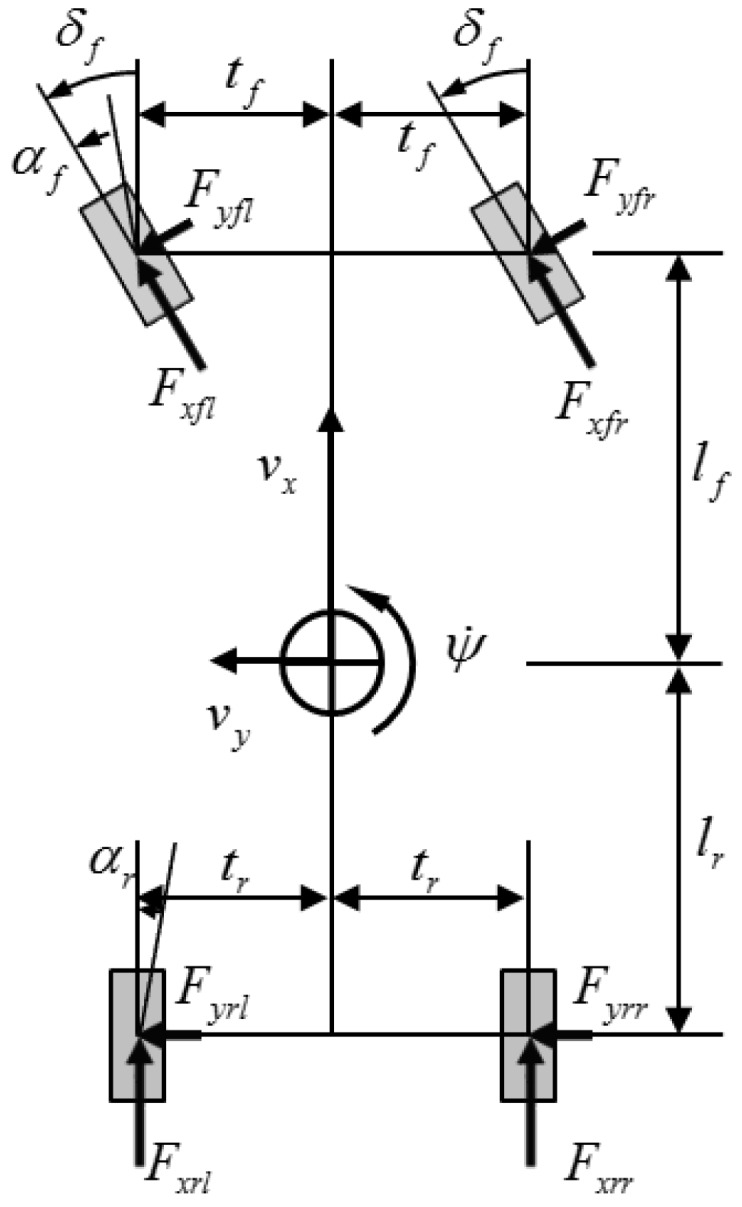
Planar two-track model.

A planar two-track non-linear model, which displays the longitudinal and lateral forces of each wheel, is shown in Figure 1. From the center of gravity, the dynamics of various two-track non-linear models in a single coordinate system is developed [32,33]. However, in the current study, the dynamic equations of the longitudinal, lateral, and yaw directions are used. They are expressed as follows:

(1) $${\dot{v}}_{x}=\dot{\psi }\cdot v_{y}+\frac{1}{m}\left\{\left(F_{xfl}+F_{xfr}\right)\cos\delta_{f}+\left(F_{yfl}+F_{yfr}\right)\sin\delta_{f}+\left(F_{xrl}+F_{xrr}\right)\right\}$$

(2) $${\dot{v}}_{y}=-\dot{\psi }\cdot v_{x}+\frac{1}{m}\left\{\left(F_{xfl}+F_{xfr}\right)\sin\delta_{f}+\left(F_{yfl}+F_{yfr}\right)\cos\delta_{f}+\left(F_{yrl}+F_{yrr}\right)\right\}$$

(3) $$\begin{array}{l} I_{z}\ddot{\psi }=l_{f}\left\{\left(F_{yfl}+F_{yfr}\right)\cos\delta_{f}+\left(F_{xfl}+F_{xfr}\right)\sin\delta_{f}\right\}-l_{r}\left(F_{yrl}+F_{yrr}\right)\\ \;+t_{f}\left\{\left(F_{yfl}-F_{yfr}\right)\sin\delta_{f}+\left(-F_{xfl}+F_{xfr}\right)\cos\delta_{f}\right\}+t_{r}\left(-F_{xrl}+F_{xrr}\right) \end{array}$$

where $v_{x}$ is the longitudinal vehicle speed, $v_{y}$ is the lateral vehicle speed, $\dot{\psi }$ is the yaw rate, $F_{x}$ is the longitudinal force, $F_{y}$ is the lateral force, m is the vehicle mass, $l_{f,r}$ is the distance between the mass center and each axle (front and rear), $t_{f,r}$ is the vehicle tread, $\delta_{f}$ is the front tire steering angle, and $I_{z}$ is the yaw inertia.

### 2.2. Non-Linear Simple Tire Model

In this study, to calculate the longitudinal and lateral forces of the two-track model, the linear and non-linear intervals are simulated to be as close as possible to the actual condition while using the non-linear simple tire model, which is relatively easy to tune. The non-linear simple tire model is realized using the hyperbolic tangent function, and the mathematical equations describing the longitudinal and lateral forces are expressed as follows:

(4) $$F_{x}=k_{x}F_{z}\tanh\left(\varepsilon_{x}\kappa \right)$$

(5) $$F_{y}=k_{x}F_{z}\tanh\left(\varepsilon_{y}\alpha \right)$$

where $k_{x,y}$ and $\varepsilon_{x,y}$ refer to the tuning factors whereas κ and α refer to the longitudinal slip and tire-side slip angle, respectively.

The longitudinal slip and tire-side slip angle for each wheel are expressed as follows [34]:

(6) $$\begin{array}{l} \kappa_{fl}=\frac{r\omega_{wfl}}{v_{x}-t_{f}\dot{\psi }}-1,\;\kappa_{fr}=\frac{r\omega_{wfl}}{v_{x}+t_{f}\dot{\psi }}-1,\\ \kappa_{rl}=\frac{r\omega_{wfl}}{v_{x}-t_{r}\dot{\psi }}-1,\;\kappa_{rr}=\frac{r\omega_{wfl}}{v_{x}+t_{r}\dot{\psi }}-1 \end{array}$$

(7) $$\begin{array}{l} \alpha_{fl}=\delta_{f}-\frac{v_{y}+l_{f}\dot{\psi }}{v_{x}-t_{f}\dot{\psi }},\;\alpha_{fr}=\delta_{f}-\frac{v_{y}+l_{f}\dot{\psi }}{v_{x}+t_{f}\dot{\psi }},\\ \alpha_{rl}=-\frac{v_{y}-l_{r}\dot{\psi }}{v_{x}-t_{r}\dot{\psi }},\;\alpha_{rr}=-\frac{v_{y}-l_{r}\dot{\psi }}{v_{x}+t_{r}\dot{\psi }} \end{array}$$

where $\omega_{w}$ is the wheel velocity.

In addition, vertical force $F_{z}$ is obtained using weight transfer, which is calculated using the longitudinal and lateral accelerations. The results are expressed as follows [34]:

(8) $$\begin{array}{l} F_{zfl}=\frac{mgl_{r}}{2l}-\frac{m_{s}a_{x}h_{s}}{2l}-k_{f}\frac{m_{s}a_{y}h_{s}}{t_{f}},\;F_{zfr}=\frac{mgl_{r}}{2l}-\frac{m_{s}a_{x}h_{s}}{2l}+k_{f}\frac{m_{s}a_{y}h_{s}}{t_{f}},\\ F_{zrl}=\frac{mgl_{f}}{2l}+\frac{m_{s}a_{x}h_{s}}{2l}-k_{r}\frac{m_{s}a_{y}h_{s}}{t_{r}},\;F_{zrr}=\frac{mgl_{f}}{2l}+\frac{m_{s}a_{x}h_{s}}{2l}+k_{r}\frac{m_{s}a_{y}h_{s}}{t_{r}} \end{array}$$

where $m_{s}$ is the vehicle sprung mass, l is the wheel base, $h_{s}$ is the sprung mass height, $t_{f,r}$ is the vehicle tread, $a_{x}$ is the longitudinal acceleration, $a_{y}$ is the lateral acceleration, $k_{f}$ is the lateral weight-shift distribution on the front wheel, and $k_{r}$ is the lateral weight-shift distribution on the rear wheel.

**Figure 2:**
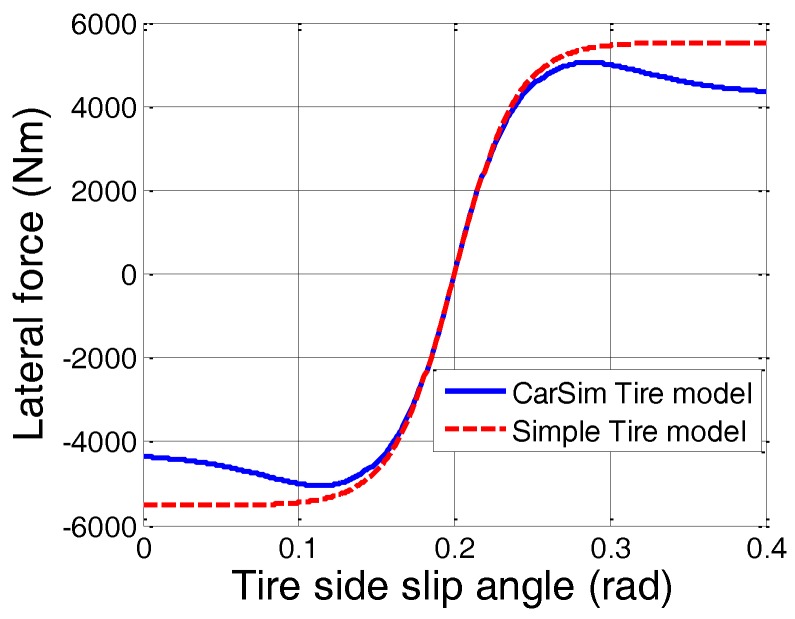
Comparison of a simple tire model with the Carsim tire model.

Figure 2 shows the comparison of the CarSim and simple tire models described in Equation (5). CarSim is a commercially available simulation tool that predicts the performance of vehicles in response to driver controls in a given environment [35,36,37].

### 2.3. Wheel Dynamics

Using the motor drive torque and wheel velocity, we can express the wheel dynamics as follows:

(9) $${\dot{\omega }}_{wi}=\frac{1}{I_{\omega }}\left(T_{mi}-r_{eff}F_{xi}-M_{yrri}\right),\;i=fl,fr,rl,rr$$

where $T_{m}$ refers to the motor drive torque, $M_{yrr}$ refers to the rolling resistance, $r_{eff}$ refers to the effective rolling radius, and $I_{\omega }$ refers to the tire inertial moment.

### 2.4. Residual

Residual is a model error. In an ideal case, the residual should only be influenced by the faults to be detected.

The residual, which includes the target for diagnosis $T_{m}$, can be expressed as follows:

(10) $$\begin{array}{ccc} r_{i}: & 0=\frac{1}{I_{\omega }}\left(T_{mi}-r_{eff}F_{xi}-M_{yrri}\right)-{\dot{\omega }}_{wi,measure}, & i=1,2,3,4=fl,fr,rl,rr \end{array}.$$

$M_{yrr}$ is practically difficult to calculate. However, $M_{yrri}$ can be ignored using an adaptive residual threshold under the assumption that the size of $M_{yrr}$ is less than the other components when the motor is driven.

### 2.5. Longitudinal Force Estimation

When Equation (10) is examined in terms of the residual, $F_{x}$, which is calculated from the model, is used. However, in the linear interval of $F_{x}$, the gradient differs depending on the terrain type. Therefore, calculation of an exact value is difficult. Hence, using the longitudinal dynamics and the non-linear simple tire model, gradient coefficient $k_{x}$ in the linear interval of $F_{x}$ can be estimated to calculate $F_{x}$ more accurately.

Henceforth, we assume that the signs of the longitudinal slips of each wheel while driving are the same and that the longitudinal force is larger than the lateral force.

The expanded longitudinal dynamics without considering lateral force is expressed as follows:

(11) $$F_{x,total}=\left(F_{xfl}+F_{xfr}\right)\cos\delta_{f}+\left(F_{xrl}+F_{xrr}\right)=m\left({\dot{v}}_{x}-\dot{\psi }\cdot v_{y}\right).$$

Using the non-linear simple tire model, expanded $F_{x,total}$ in Equation (11) can be expressed as follows:

(12) $$m\left({\dot{v}}_{x}-\dot{\psi }\cdot v_{y}\right)=k_{x}\left\{\begin{array}{l} F_{zfl}\tanh\left(\varepsilon_{x}\kappa_{fl}\right)\cos\delta_{f}+F_{zfr}\tanh\left(\varepsilon_{x}\kappa_{fr}\right)\cos\delta_{f}\\ +F_{zrl}\tanh\left(\varepsilon_{x}\kappa_{rl}\right)+F_{zrr}\tanh\left(\varepsilon_{x}\kappa_{rr}\right) \end{array}\right\}.$$

In the linear interval of $F_{x}$, gradient coefficient $k_{x}$ can be expressed as follows by rearranging the terms in Equation (12):

(13) $$k_{x}=\frac{m\left({\dot{v}}_{x}-\dot{\psi }\cdot v_{y}\right)}{\left\{\begin{array}{l} F_{zfl}\tanh\left(\varepsilon_{x}\kappa_{fl}\right)\cos\delta_{f}+F_{zfr}\tanh\left(\varepsilon_{x}\kappa_{fr}\right)\cos\delta_{f}\\ +F_{zrl}\tanh\left(\varepsilon_{x}\kappa_{rl}\right)+F_{zrr}\tanh\left(\varepsilon_{x}\kappa_{rr}\right) \end{array}\right\}}.$$

With $k_{x}$, a more accurate longitudinal force can be estimated to increase the accuracy of the residual fault diagnosis. Hence, the longitudinal force can be written as

(14) $$F_{xi}=k_{x}F_{zi}\tanh\left(\varepsilon_{x}\kappa_{i}\right),\;i=fl,fr,rl,rr.$$

### 2.6. Analysis of the Correlation between Each Sensor and the Residual

To configure the fault-diagnosis algorithm and to confirm the possibility of fault separation, the correlation between each sensor and residual needs to be analyzed.

The sensor information used for estimating the longitudinal force is expressed as follows:

(15) $$\begin{array}{l} \begin{array}{ccc} c_{1}: & F_{xi}=c_{1}\left(\delta_{f},\dot{\psi },v_{x},v_{y},F_{z},\kappa_{i}\right),\; & i=1,2,3,4=fl,fr,rl,rr \end{array}\\ \;\kappa_{i}=c_{1}\prime (\dot{\psi },v_{x},\omega_{wi}) \end{array}.$$

Equation (15) indicates that $F_{x}$ is obtained using sensor signals $\delta_{f}$, $\dot{\psi }$, $v_{x}$, $v_{y}$, $F_{z}$, and $\omega_{w}$.

$v_{y}$ in Equation (15) can be obtained using Equations (2) and (5), whereas $F_{z}$ can be obtained using Equation (8), i.e.,

(16) $$\begin{array}{cc} c_{2}: & v_{y}=c_{2}\left(\delta_{f},\dot{\psi },v_{x},F_{x},F_{y}\right) \end{array}$$

(17) $$\begin{array}{cc} c_{3}: & F_{y}=c_{3}\left(\alpha_{i},F_{z}\right) \end{array}$$

(18) $$\begin{array}{cc} c_{4}: & F_{z}=c_{4}\left(a_{x},a_{y}\right) \end{array}$$

Finally, the correlation between each sensor and the residual can be expressed as follows using Equation (10):

(19) $$\begin{array}{lll} r_{i}: & 0=r_{i}\left(T_{mi},F_{xi},\omega_{w}\right)=r_{i}\left(T_{mi},a_{x},a_{y},\delta_{f},\dot{\psi },v_{x},\omega_{w}\right), & i=fl,fr,rl,rr=1,2,3,4 \end{array}$$

**Figure 3:**
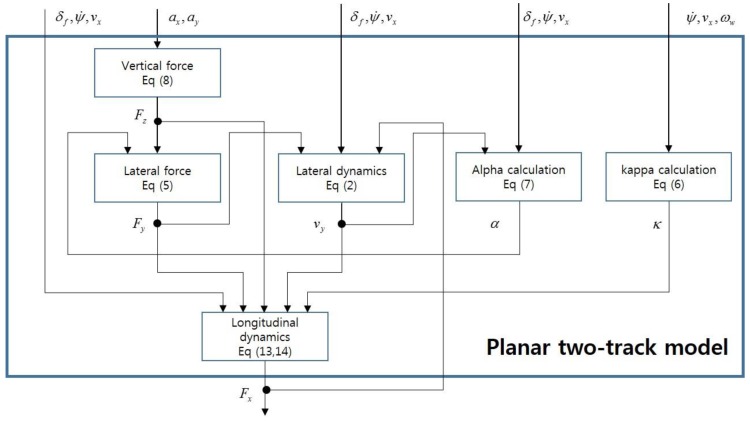
Sensor information used for estimating the longitudinal force.

Figure 3 shows the correlation between each sensor and the residual.

The residuals obtained from Equation (19) and Figure 3 can be independently configured for each wheel and are listed as follows Table 1.

The term “X” in Table 1 refers to the correlation between the residual $r_{i}(i=1\sim 4)$ and each sensor. In other words, by assuming that the other sensor information is normal, the fault of the drive motor $T_{m}$ in each wheel can be detected and separated. If dynamic sensors such as the yaw-rate, longitudinal and lateral acceleration, and wheel-speed sensors, fail, the residual $r_{i}(i=1\sim 4)$ cannot diagnose the motor sensor fault. Therefore, for the fault separation in the other sensors, fault residual redundancy is required. In this study, Equations (2), (3) and (5) are used to add the residuals of $\dot{\psi }$ and $v_{y}$ while obtaining the residual of $v_{x}$ using the Global Positioning System (GPS). In addition, using Equation (4), the residual of the longitudinal force can be substituted.

In order to isolate motor sensor faults and vehicle dynamics sensor faults, additional residuals should not be affected by the fault of the drive motor $T_{m}$. Therefore, it is necessary to separate the vehicle dynamics sensor faults using a linear model instead of a complex model.

Using the linear bicycle model [38], the estimations of $\dot{\psi }$ and $v_{y}$ can be expressed as follows:

(20) $$\left[\begin{array}{c} {\dot{v}}_{y,est}\\ {\ddot{\psi }}_{est} \end{array}\right]=\left[\begin{array}{cc} -\frac{C_{\alpha f}+C_{\alpha r}}{mv_{x}} & \frac{-C_{\alpha f}l_{f}+C_{\alpha r}l_{r}}{mv_{x}}-v_{x}\\ -\frac{C_{\alpha f}l_{f}-C_{\alpha r}l_{r}}{I_{z}v_{x}} & -\frac{C_{\alpha f}{l_{f}}^{2}+C_{\alpha r}{l_{r}}^{2}}{I_{z}v_{x}} \end{array}\right]\left[\begin{array}{c} v_{y,est}\\ {\dot{\psi }}_{est} \end{array}\right]+\left[\begin{array}{c} \frac{C_{\alpha f}}{m}\\ \frac{l_{f}C_{\alpha f}}{I_{z}} \end{array}\right]\delta_{f}$$

where $C_{\alpha f}$ and $C_{\alpha r}$ are cornering stiffness ($F_{y}=C_{\alpha }\alpha$).

Using Equation (20), the residual can be substituted as follows:

(21) $$\begin{array}{cc} r_{5}: & 0=\dot{\psi }- \end{array}{\dot{\psi }}_{est}=r_{5}(\dot{\psi },\delta_{f},v_{x})$$

Using the acceleration sensor dynamics [39] and Equation (20), the residual can be substituted as follows:

(22) $$\begin{array}{cc} r_{6}: & 0=a_{x}- \end{array}\left({\dot{v}}_{x}-{\dot{\psi }}_{est}\cdot v_{y,est}\right)=r_{6}(a_{x},\dot{\psi },\delta_{f},v_{x})$$

(23) $$\begin{array}{cc} r_{7}: & 0=a_{y}- \end{array}\left({\dot{v}}_{y,est}+{\dot{\psi }}_{est}\cdot v_{x}\right)=r_{7}(a_{y},\dot{\psi },\delta_{f},v_{x})$$

The equation substituted by the residual of $v_{x}$ using GPS is expressed as follows:

(24) $$\begin{array}{cc} r_{8}: & 0=v_{x}- \end{array}v_{x,GPS}=r_{8}(v_{x},v_{x,GPS})$$

In order to distinguish between wheel sensor failure and motor failure, the additional residual is configured as wheel dynamics only.

Assuming $k_{x}$ is a known value, Equation (4) can be expressed as

(25) $$\begin{array}{ll} c_{9}: & F_{xfl,2}=c_{9}(F_{z},\dot{\psi },v_{x},\omega_{fl})\\ c_{10}: & F_{xfr,2}=c_{10}(F_{z},\dot{\psi },v_{x},\omega_{fr})\\ c_{11}: & F_{xrl,2}=c_{11}(F_{z},\dot{\psi },v_{x},\omega_{rl})\\ c_{12}: & F_{xrr,2}=c_{12}(F_{z},\dot{\psi },v_{x},\omega_{rr}) \end{array}$$

In addition, Equation (25) can also be substituted by the residuals using Equation (19), as shown below.

(26) $$\begin{array}{cc} r_{9}: & 0=r_{9}(T_{mfl},F_{xfl,2},\omega_{fl})=r_{9}(T_{mfl},c_{9}(c_{1}(a_{x},a_{y}),\dot{\psi },v_{x},\omega_{fl}),\omega_{fl}) \end{array}$$

(27) $$\begin{array}{cc} r_{10}: & 0=r_{10}(T_{mfr},F_{xfr,2},\omega_{fr})=r_{10}(T_{mfr},c_{10}(c_{1}(a_{x},a_{y}),\dot{\psi },v_{x},\omega_{fr}),\omega_{fr}) \end{array}$$

(28) $$\begin{array}{cc} r_{11}: & 0=r_{11}(T_{mrl},F_{xrl,2},\omega_{rl})=r_{11}(T_{mrl},c_{11}(c_{1}(a_{x},a_{y}),\dot{\psi },v_{x},\omega_{rl}),\omega_{rl}) \end{array}$$

(29) $$\begin{array}{cc} r_{12}: & 0=r_{12}(T_{mrr},F_{xrr,2},\omega_{rr})=r_{12}(T_{mrr},c_{12}(c_{1}(a_{x},a_{y}),\dot{\psi },v_{x},\omega_{rr}),\omega_{rr}) \end{array}$$

When the residuals derived from the above are summarized, $r_{i}(i=1\sim 4)$ were diagnosed using a planar two-track model to detect the failure of each wheel motor. $r_{i}(i=5\sim 8)$ were diagnosed using a linear bicycle model to diagnose vehicle dynamics failure without being affected by motor failure. $r_{i}(i=9\sim 12)$ are additional residuals that are used to separate the wheel sensor and motor fault.

Residuals that are newly added to the list in Table 1 are listed in Table 2.

Table 2 confirms that the other sensors are also capable of separating the faults using additional residuals.

### 2.7. Adaptive Threshold

**Figure 4:**
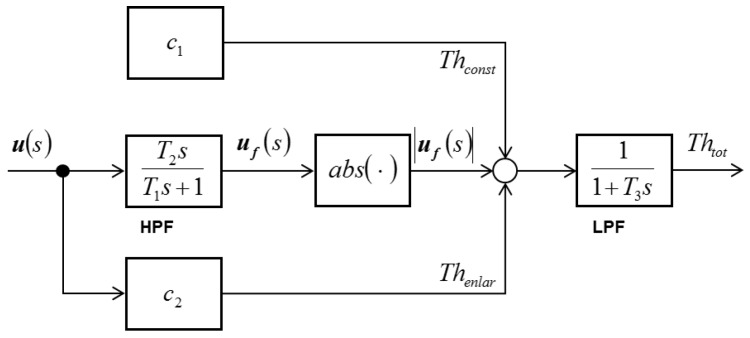
Structure of the adaptive threshold generator.

The vehicle and tire models used in the present study show behavior similar to that of actual models in a normal state. However, in a transient state, the inaccuracy of the models increases. Therefore, the value of the residual, which is designed by including the inaccuracy, can be different from zero even when no fault exists. Such residual deviation is influenced by the intensity of the input signal and frequency. Therefore, to realize a fault-diagnosis algorithm that is robust against model inaccuracy, the method of adaptive threshold is used. Figure 4 shows the generation of adaptive threshold values according to the input value [40].

The fault-diagnosis algorithm uses vehicle models that do not fully correspond with the real processes due to model uncertainties. The generated residual then deviates from zero even without a fault. If the threshold is not well set, it may generate false alarms through normal fluctuations of the variable. Obviously, setting Th too high reduces the sensitivity to faults, and setting Th too low increases the false alarm rate. Usually, Th is empirically set by considering the maximum influence of the model uncertainties. In particular, in the transient state, these model uncertainties more frequently occur. Therefore, adaptive threshold is introduced to avoid these problems. The deviation in the residual depends on the amplitude and frequencies of the input excitation. The adaptive threshold method uses its variation. It uses a high-pass filter to enlarge the threshold (where the deviation and amplitude of the input have an effect) and a low-pass filter to smoothen the threshold, as shown in Figure 3. Time constants $T_{1}$ and $T_{3}$ are selected according to the dominant time constant of the system process. $T_{1}/T_{2}$ depends on the model uncertainty of the dynamics [40].

### 2.8. Simulation Result

To verify the proposed fault-diagnosis algorithm, Carsim and Matlab/Simulink are used. Among the vehicle models available in Carsim, “E-Class sedan” is considered as the subject. The simulation is conducted at a starting velocity of 20 km/h on a straight line with the throttle set at constant values of 0.2 and 0.5.

During the simulation, the Motor fault signal is applied at an interval of 5–7 s. The fault signal triggers reduction in the torque in the rear right (RR) wheel drive motor by 30%.

**Figure 5:**
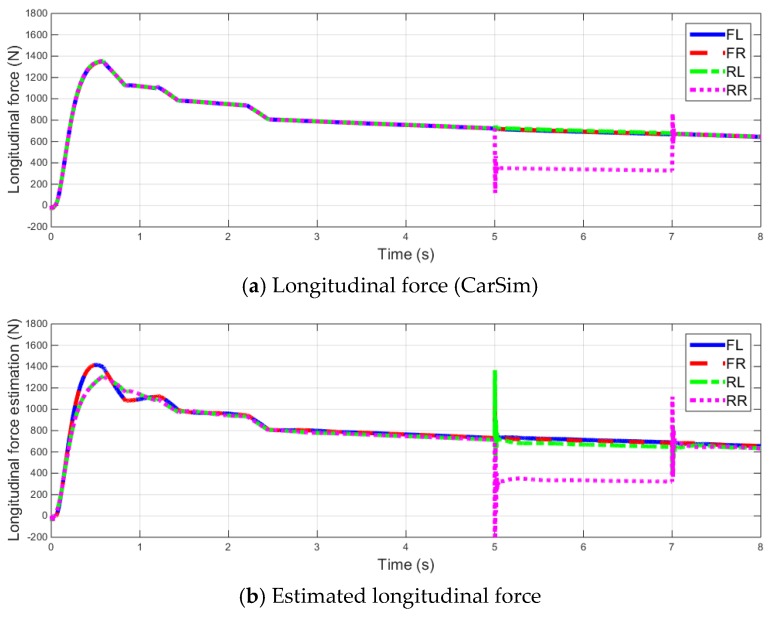

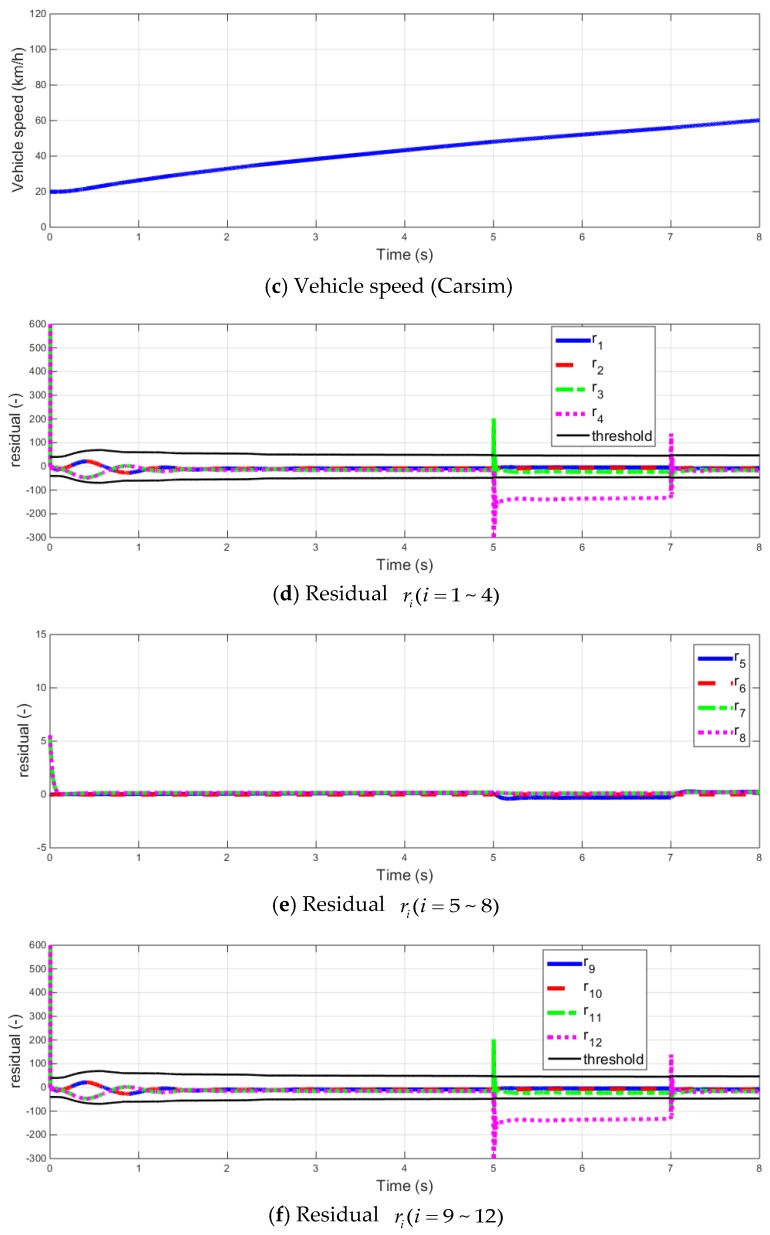
Simulation result (Initial speed 20 km/h, throttle = 0.2 acceleration).

From the simulation results shown in Figure 5, we can confirm that the estimated longitudinal forces of each wheel, derived from the longitudinal force equation and simple tire model, are almost similar to the actual Carsim value. As the longitudinal wheel slip increases, the residual also increases; therefore, the threshold value can also be set high, which proves the robustness of the model. The fault-diagnosis results according to a fault application from 5 to 7 s can also be separated.

The following simulations were performed by adding vehicle dynamics sensors faults.

The fault signal is applied from 1 to 1.5 s to the yaw rate sensor with an offset of 0.05 rad/s. An offset of g (=9.8 m/s) for the longitudinal dynamics sensor is applied from 2 to 2.5 s, and an offset of g for the lateral dynamics sensor is applied from 3 to 3.5 s. The fault signal of the front left-wheel speed sensor is applied from 4 to 4.5 s with an offset of 1 km/h.

**Figure 6:**
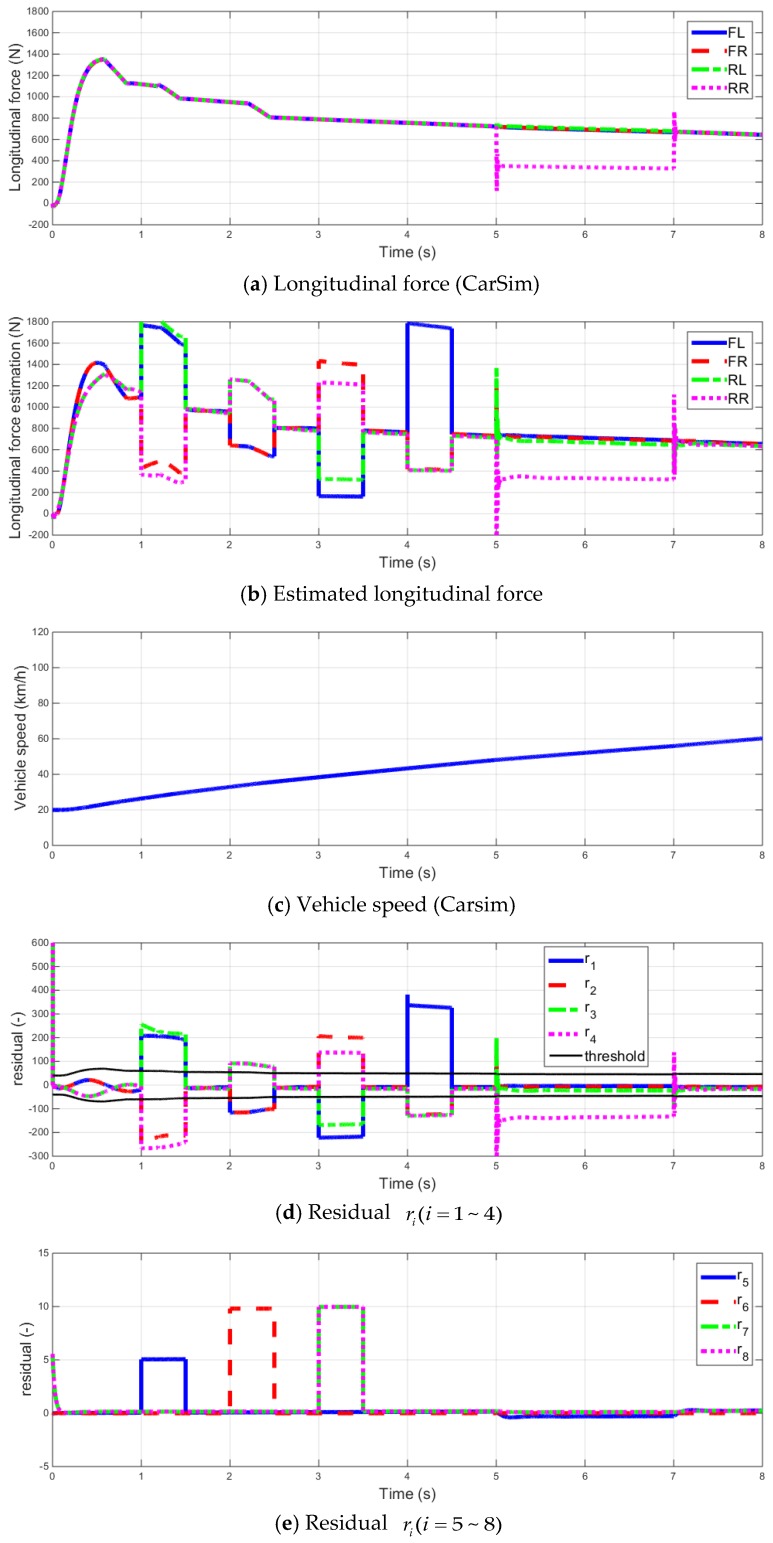

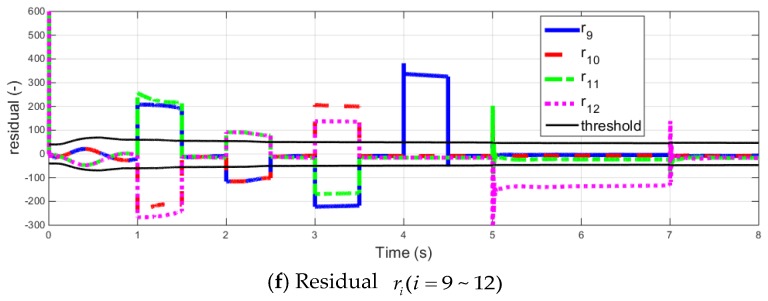
Simulation result (Initial speed 20 km/h, throttle = 0.2 acceleration, with vehicle dynamics sensor faults).

From the simulation results shown in Figure 6, when vehicle dynamic sensors fail, the estimated longitudinal forces of each wheel are influenced by faults. Similarly, the residual $r_{i}(i=1\sim 4)$ of fault diagnosis algorithm in [30,31] cannot diagnose faults. However, the additional residuals enable diagnosis even in the case of vehicle dynamics sensor faults through Table 2.

## 3. Low-Level Fault Diagnosis

The drive motor for an electric vehicle should satisfy various performance requirements, including structural robustness, high power and torque density, wide operating velocity range, excellent environmental resistance (seismic, heat, and corrosion), and highly efficient driving control, considering the driving characteristics. IPMSM is one of the motors that satisfy the above requirements. The IPMSM is structurally stable because a permanent magnet is built inside the rotor, and it has excellent magnetic saliency, thus possessing a weak field control for a wide range of operating velocities. In addition, its power and torque density are excellent, which enable highly efficient driving [41,42]. Therefore, in the present study, the IPMSM is chosen as the drive motor of the in-wheel independent drive electric vehicle used in the fault-diagnosis experiments.

### 3.1. IPMSM Model

**Figure 7:**
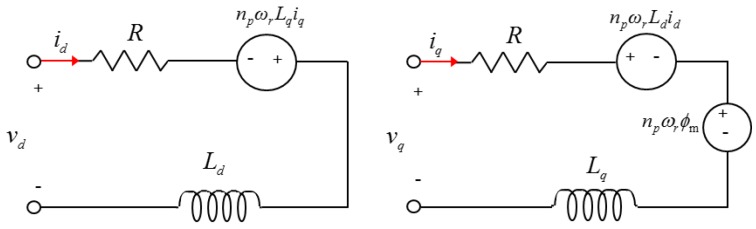
*d-q* equivalent circuit for an interior permanent magnet synchronous motor (IPMSM).

Figure 7 shows the *d*–*q* axis equivalent circuit of the IPMSM, whereas Equation (30) expresses the voltage equation of the *d*–*q* axis of the rotating coordinate system of the IPMSM.

(30) $$\begin{array}{l} v_{d}=Ri_{d}+L_{d}\frac{di_{d}}{dt}-n_{p}\omega_{r}L_{q}i_{q}\\ v_{q}=Ri_{q}+L_{q}\frac{di_{q}}{dt}+n_{p}\omega_{r}L_{d}i_{d}+n_{p}\omega_{r}\varphi_{m} \end{array}$$

where $v_{d}$ and $v_{q}$ are the *d*–*q* axis applied voltages, $i_{d}$ and $i_{q}$ are the *d*–*q* axis currents, $\omega_{r}$ is the rotor speed, R is the armature winding resistance, $L_{d}$ and $L_{q}$ are the *d*–*q* axis inductances, and $\varphi_{m}$ is the magnetic flux linkage.

Equation (31) shows the conversion of the three-phase fixed coordinate system into a two-phase rotating coordinate system.

(31) $$\left[\begin{array}{c} i_{d}\\ i_{q} \end{array}\right]=\left[\begin{array}{cc} \sin\theta & -\cos\theta \\ \cos\theta & \sin\theta \end{array}\right]\frac{2}{3}\left[\begin{array}{ccc} 1 & -\frac{1}{2} & -\frac{1}{2}\\ 0 & \frac{\sqrt{3}}{2} & -\frac{\sqrt{3}}{2} \end{array}\right]\left[\begin{array}{c} i_{a}\\ i_{b}\\ i_{c} \end{array}\right]$$

In a three-phase motor system, in most cases, owing to their costs, two-phase electric current sensors are used instead of three-phase electric current sensors. Using the current balance equation $i_{a}+i_{b}+i_{c}=0$ to delete term $i_{c}$ from Equation (31) yields the following result:

(32) $$\left[\begin{array}{c} i_{d}\\ i_{q} \end{array}\right]=\left[\begin{array}{cc} \sin\theta & -\cos\theta \\ \cos\theta & \sin\theta \end{array}\right]\left[\begin{array}{cc} 1 & 0\\ \frac{\sqrt{3}}{3} & \frac{2\sqrt{3}}{3} \end{array}\right]\left[\begin{array}{c} i_{a}\\ i_{b} \end{array}\right].$$

### 3.2. Current and Position Sensor Fault Diagnosis

The fault of the electric current sensor can be diagnosed using the parity equation [43]. The space equation to obtain the parity equation is expressed by Equation (33).

(33) $$\begin{array}{l} \dot{x}=Ax+Bu+E_{x}d+F_{x}f\\ y=Cx+Du+E_{y}d+F_{y}f \end{array}$$

where $x\in {ℜ}^{n}$ denotes the state vector, $u\in {ℜ}^{m}$ is the vector of the measured input signals, $y\in {ℜ}^{p}$ is the vector of the measured plant output signals, and $d\in {ℜ}^{n_{d}}$ and $f\in {ℜ}^{n_{f}}$ are vectors of unknown input signals. f represents the faults one desires to detect, whereas d represents unknown disturbances that should not be detected.

When used as a transfer equation, Equation (33) can be converted to

(34) $$y\left(s\right)=H_{yu}\left(s\right)u\left(s\right)+H_{yx}\left(s\right)x\left(0\right)+H_{yd}\left(s\right)d\left(s\right)+H_{yf}\left(s\right)f\left(s\right)$$

$$\mathrm{where}\;\{\begin{array}{l} H_{yu}\left(s\right)=C{\left(sI-A\right)}^{-1}B+D\\ H_{yx}\left(s\right)=C{\left(sI-A\right)}^{-1}\\ H_{yd}\left(s\right)=C{\left(sI-A\right)}^{-1}E_{x}+E_{y}\\ H_{yf}\left(s\right)=C{\left(sI-A\right)}^{-1}F_{x}+F_{y} \end{array}.$$

The residual can be obtained by the algorithm shown in Figure 7 using the transfer function expressed in Equation (34).

**Figure 8:**
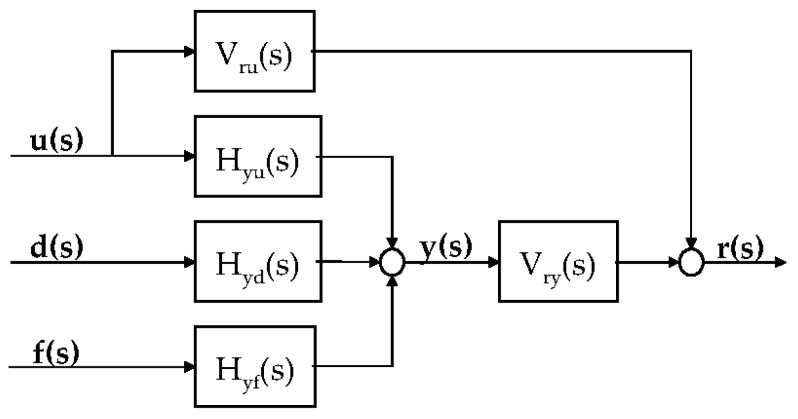
Structure of a residual generator using the parity equation.

When the residual shown in Figure 8 is mathematically expressed, the result is expressed as Equation (35). The residual can be obtained from the difference between the two vectors, which are obtained by multiplying the input and output vectors with design vectors $V_{ru}\left(s\right)$ and $V_{ry}\left(s\right)$, respectively. The residual r(s) is independent of the known input vector but depends on the fault vector [32].

(35) $$\begin{array}{l} r\left(s\right)=V_{ru}\left(s\right)u\left(s\right)+V_{ry}\left(s\right)y\left(s\right)\\ \;=V_{ru}\left(s\right)u\left(s\right)+V_{ry}\left(s\right)\left\{H_{yu}\left(s\right)u\left(s\right)+H_{yd}\left(s\right)d\left(s\right)+H_{yx}\left(s\right)x\left(0\right)+H_{yf}\left(s\right)f\left(s\right)\right\}\\ \;=\left[\begin{array}{cc} V_{ru}\left(s\right)+V_{ry}\left(s\right)H_{yu}\left(s\right) & V_{ry}\left(s\right)H_{yd}\left(s\right) \end{array}\right]\left[\begin{array}{c} u\left(s\right)\\ d\left(s\right) \end{array}\right]\\ \;+V_{ry}\left(s\right)H_{yx}\left(s\right)x\left(0\right)+V_{ry}\left(s\right)H_{yf}\left(s\right)f\left(s\right) \end{array}$$

To create a residual that is influenced only by the fault signal, the coefficients of u(s) and d(s) should be zero. Therefore, $V_{ru}\left(s\right)$ and $V_{ry}\left(s\right)$ that satisfy Equation (36) should be obtained.

(36) $$\left[\begin{array}{cc} V_{ry} & V_{ru} \end{array}\right]\left[\begin{array}{cc} H_{yu} & H_{yd}\\ I & 0 \end{array}\right]=0$$

When the above method is applied to the IPMSM, the basic form of Equation (35) can be expressed as follows:

(37) $$\begin{array}{l} \dot{x}=Ax+Bu+E_{x}d\\ y=Cx+Du+E_{y}d+F_{y}f\\ x=\left[\begin{array}{c} i_{d}\\ i_{q} \end{array}\right],u=\left[\begin{array}{c} v_{d}\\ v_{q}-n_{p}\omega_{r}\varphi_{m} \end{array}\right],y=\left[\begin{array}{c} i_{a}\\ i_{b} \end{array}\right],f=\left[\begin{array}{c} i_{a\_f}\\ i_{b\_f} \end{array}\right]\\ A=\left[\begin{array}{cc} -\frac{R}{L_{d}} & \frac{n_{p}\omega_{r}L_{q}}{L_{d}}\\ -\frac{n_{p}\omega_{r}L_{d}}{L_{q}} & -\frac{R}{L_{q}} \end{array}\right],B=\left[\begin{array}{cc} \frac{1}{L_{d}} & 0\\ 0 & \frac{1}{L_{q}} \end{array}\right]\\ C=\left[\begin{array}{cc} \sin\theta & -\cos\theta \\ \cos\theta & \sin\theta \end{array}\right]\left[\begin{array}{cc} 1 & 0\\ \frac{\sqrt{3}}{3} & \frac{2\sqrt{3}}{3} \end{array}\right],D=0\\ F_{y}=\left[\begin{array}{cc} \sin\theta & -\cos\theta \\ \cos\theta & \sin\theta \end{array}\right]\left[\begin{array}{cc} 1 & 0\\ \frac{\sqrt{3}}{3} & \frac{2\sqrt{3}}{3} \end{array}\right] \end{array}.$$

We assume that only the fault of the electric current sensor is considered and that the disturbance is low ($E_{x}=E_{y}=0$). Equation (37) is converted into the form of a transfer function similar to Equation (35) when we assume that $\omega_{r}$ is a pseudo constant [44].

Then, the resulting equation would be as follows:

(38) $$\begin{array}{l} H_{yu}(s)=\left[\begin{array}{cc} \sin\theta & -\cos\theta \\ \cos\theta & \sin\theta \end{array}\right]\left[\begin{array}{cc} 1 & 0\\ \frac{\sqrt{3}}{3} & \frac{2\sqrt{3}}{3} \end{array}\right]\frac{1}{K}\left[\begin{array}{cc} R+sL_{q} & n_{p}\omega_{r}L_{q}\\ -n_{p}\omega_{r}L_{d} & R+sL_{q} \end{array}\right]\\ H_{yd}(s)=0 \end{array}$$

where $K=(R+sL_{d})(R+sL_{q})+{n_{p}}^{2}{\omega_{r}}^{2}L_{d}L_{q}$.

When Equation (38) is applied to Equation (37), $V_{ry}(s)$ and $V_{ru}(s)$ can be obtained as follows:

(39) $$\begin{array}{l} V_{ry}(s)=\left[\begin{array}{cc} -n_{p}\omega_{r}L_{d} & -R-sL_{q}\\ -R-sL_{d} & n_{p}\omega_{r}L_{q} \end{array}\right]\\ V_{ru}(s)=\left[\begin{array}{cc} 0 & 1\\ 1 & 0 \end{array}\right] \end{array}.$$

Finally, the residual derivation, i.e., Equation (39), can be used to derive the following:

(40) $$\begin{array}{l} r\left(s\right)=V_{\mathrm{ry}}\left(s\right)y\left(s\right)+V_{\mathrm{ru}}\left(s\right)u\left(s\right)\\ \;=\left[\begin{array}{cc} -n_{p}\omega_{r}L_{d} & -R-sL_{q}\\ -R-sL_{d} & n_{p}\omega_{r}L_{q} \end{array}\right]y\left(s\right)+\left[\begin{array}{cc} 0 & 1\\ 1 & 0 \end{array}\right]u\left(s\right)\\ \;=\left[\begin{array}{cc} -n_{p}\omega_{r}L_{d} & -R-sL_{q}\\ -R-sL_{d} & n_{p}\omega_{r}L_{q} \end{array}\right]\left[\begin{array}{cc} \sin\theta & -\cos\theta \\ \cos\theta & \sin\theta \end{array}\right]\left[\begin{array}{cc} 1 & 0\\ \frac{\sqrt{3}}{3} & \frac{2\sqrt{3}}{3} \end{array}\right]f(s) \end{array}.$$

Now, coordinate conversion will be conducted to independently separate the residual from the faults in the electric current sensor at *a* and *b*.

(41) $$\begin{array}{l} r^{\prime }\left(s\right)={\left(\left[\begin{array}{cc} -n_{p}\omega_{r}L_{d} & -R-sL_{q}\\ -R-sL_{d} & n_{p}\omega_{r}L_{q} \end{array}\right]\left[\begin{array}{cc} \sin\theta & -\cos\theta \\ \cos\theta & \sin\theta \end{array}\right]\left[\begin{array}{cc} 1 & 0\\ \frac{\sqrt{3}}{3} & \frac{2\sqrt{3}}{3} \end{array}\right]\right)}^{-1}r\left(s\right)\\ \;=\left[\begin{array}{cc} 1 & 0\\ 0 & 1 \end{array}\right]f(s)\\ \;=\left[\begin{array}{c} r_{13}\\ r_{14} \end{array}\right] \end{array}$$

From the above mathematical expressions, we confirm that residuals $r_{13}$ and $r_{14}$ are influenced by the faults of electric current sensors $i_{a\_f}$ and $i_{b\_f}$, respectively.

Because the fault-diagnosis algorithm mentioned above considers only the fault of the electric current sensor, to confirm the possibility of fault separation regarding the position sensor of the residual in Equation (31), the correlation between each sensor and the residual will be analyzed.

Using Equations (40) and (41), the sensor information that influences $r_{13}$ and $r_{14}$ can be expressed as follows:

(42) $$r_{13}:0=r_{13}(i_{a},\theta )$$

(43) $$r_{14}:0=r_{14}(i_{b},\theta ).$$

The expressions for Equations (42) and (43) are listed in Table 3.

Assuming that only a single fault occurs, Table 3 lists the possibility of separation of electric current sensor faults $i_{a}$ and $i_{b}$ from fault θ of the position sensor using the combination of $r_{13}$ and $r_{14}$.

### 3.3. Simulation Result

The proposed algorithm is implemented using Matlab/Simulink. The IPMSM control system model is selected among the AC6 100-kW drive samples of Matlab/Simulink. The motor model parameters of the example are listed in Table 4.

**Figure 9:**
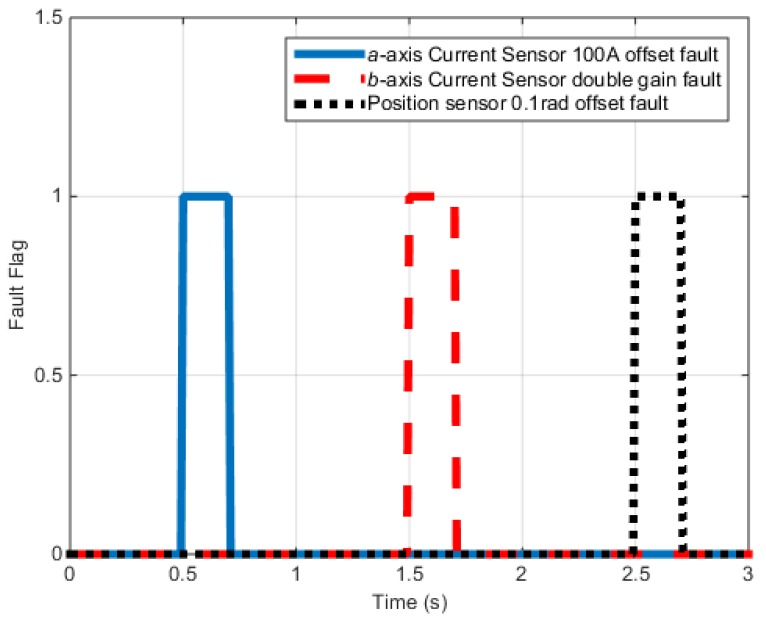
Torque control simulation results (Fault flag).

Figure 9 shows the fault signals of each sensor. The fault signal is applied from 0.5 to 0.7 s to the a-phase electric sensor with 100-A offset. From 1 to 1.2 s, the gain value of the b-phase electric sensor doubles and an offset of 0.1 rad/s of the position sensor is applied from 1.5 to 1.7 s.

**Figure 10:**
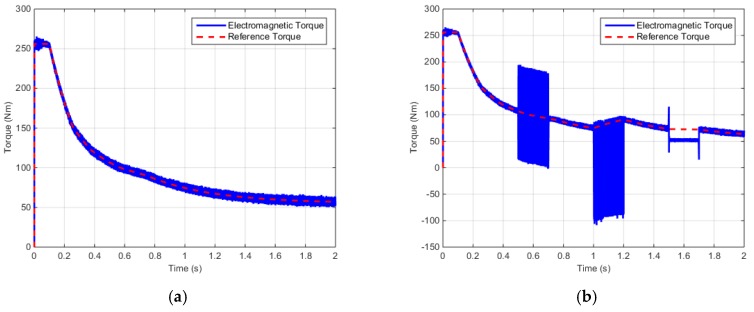
Torque control simulation results (Output torque): (**a**) Output torque (Normal); (**b**) Output torque (Faulty).

Figure 10 shows the electromagnetic torque of the IPMSM control system according to the reference torque.

**Figure 11:**
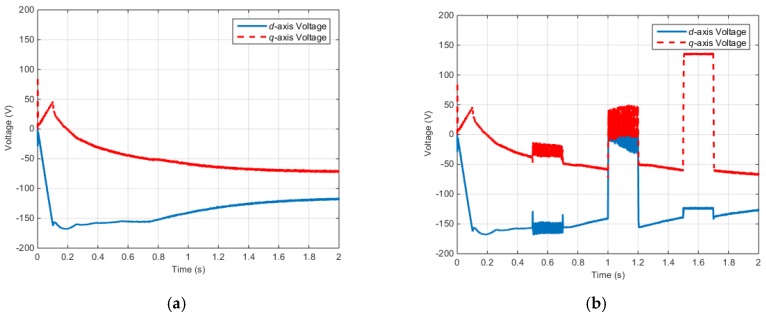
Torque control simulation results (Input voltage): (**a**) Rotor voltages (Normal); (**b**) Rotor voltages (Faulty).

Figure 11 shows the *d*–*q* axis input voltages of the IPMSM control system.

**Figure 12:**
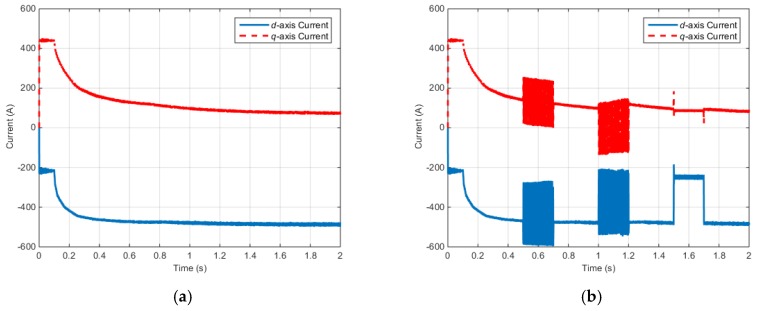
Torque control simulation results (Rotor currents): (**a**) Rotor currents (Normal); (**b**) Rotor currents (Faulty).

Figure 12 shows the *d*–*q* axis electric currents of the IPMSM control system.

According to the simulation results described above, when faults occur in the electric and positions sensor, the faults are influenced by the electromagnetic torque, input voltage, and electric current. Similarly, a fault in one part of the control system can influence the other parts.

**Figure 13:**
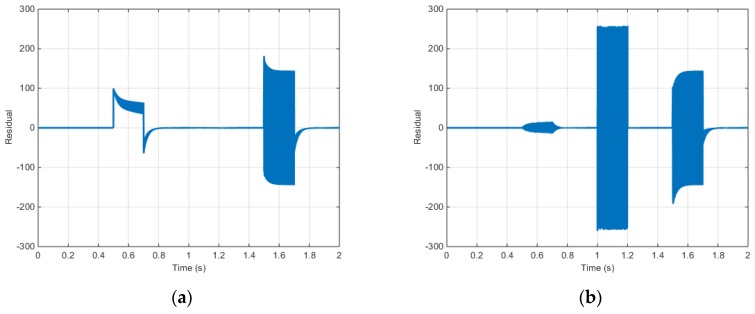
Torque control simulation results (residual): (**a**) Residual ($r_{13}$); (**b**) Residual ($r_{14}$).

Figure 13 shows $r_{13}$ and $r_{14}$ from the proposed algorithm during the fault simulation.

When Figure 13 is considered as the fault table listed in Table 3, $r_{13}$ significantly deviates from zero in case of an a-phase electric current sensor fault; meanwhile, $r_{14}$ significantly deviates from zero in case of a b-phase electric current sensor fault. In case of a position sensor fault, both $r_{13}$ and $r_{14}$ significantly deviate from zero. Assuming that only a single fault occurs, residuals $r_{13}$ and $r_{14}$ can be used to detect and separate the faults in the a- and b-phase electric current sensors and the position sensor.

### 3.4. Experimental Result

**Figure 14:**
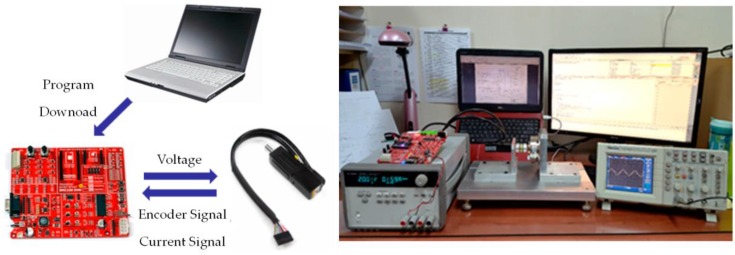
Experimental test environment.

The validity of the algorithm is verified through experiments. Figure 14 shows the test environment.

The motor parameters of the experiments are listed in Table 5.

**Figure 15:**
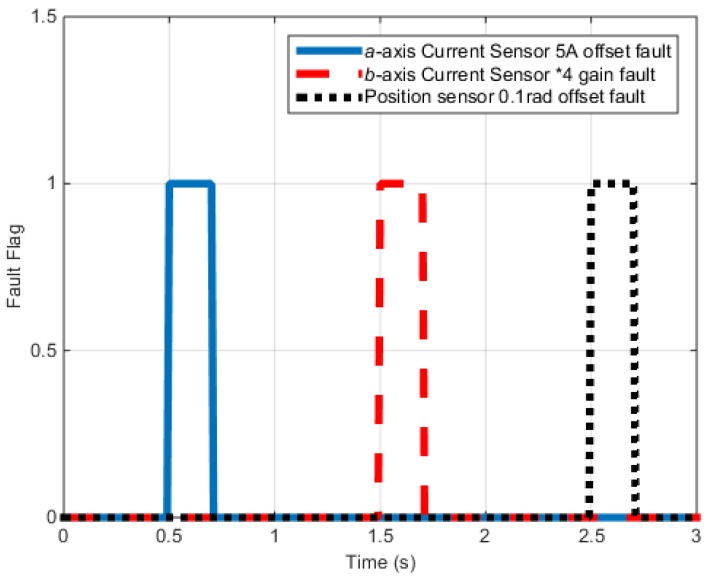
Speed control test results (Fault flag).

Figure 15 shows the fault signals of each sensor. The fault signal is applied from 0.5 to 0.7 s to the a-phase electric sensor with 5-A offset. From 1.5 to 1.7 s, the gain value of the b-phase electric sensor doubles while 0.1-rad offset is applied from 2.5 to 2.7 s.

**Figure 16:**
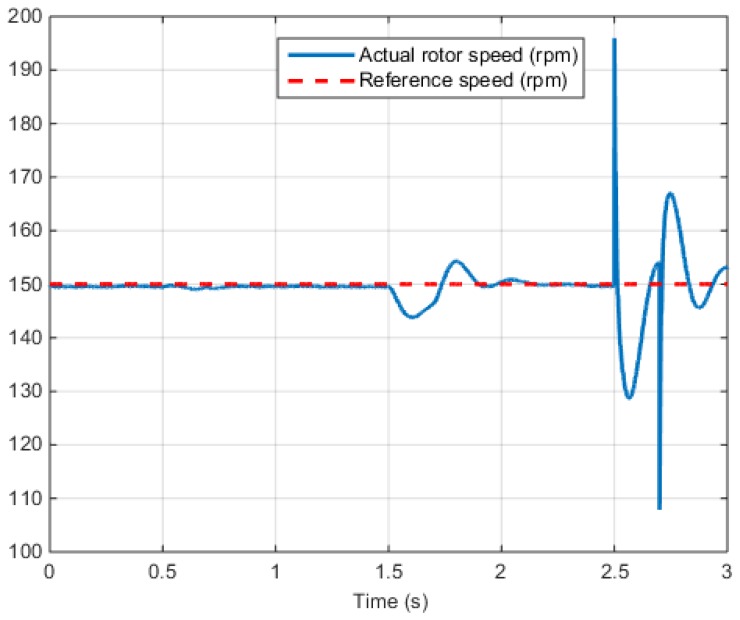
Speed control test results (Output speed).

Figure 16 shows the rotor speed of the PMSM control system according to the reference speed.

**Figure 17:**
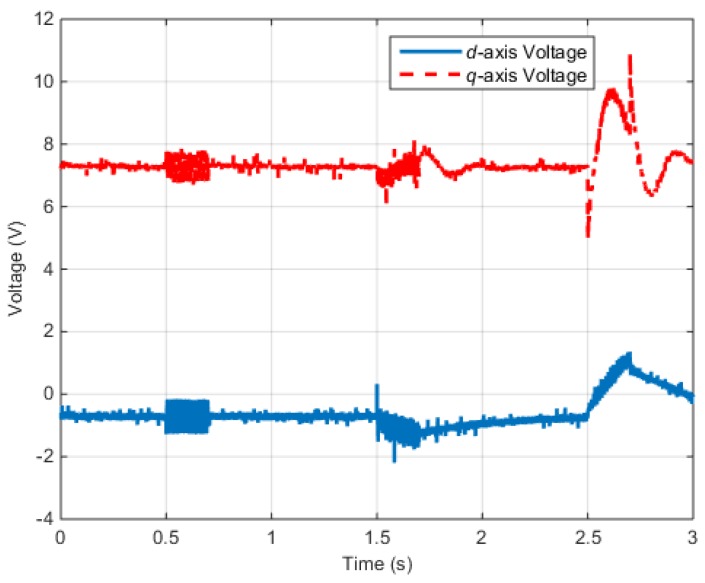
Speed control test results (Input voltage).

Figure 17 shows the *d*–*q* axis input voltages of the PMSM control system.

**Figure 18:**
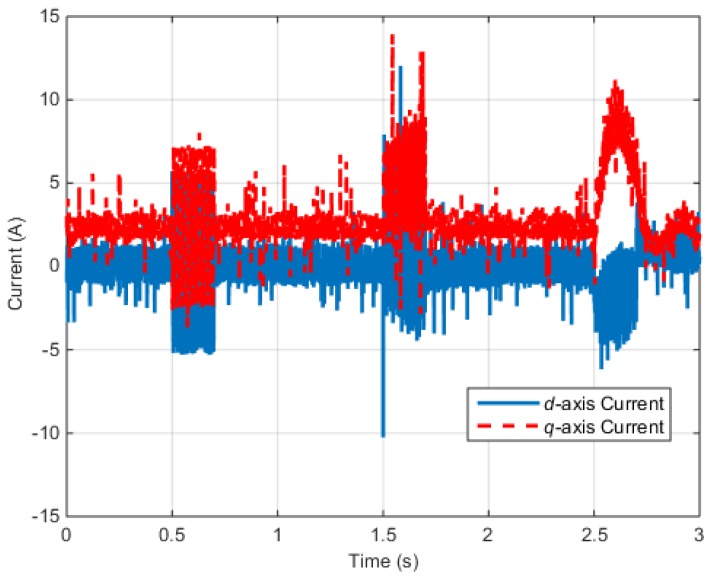
Speed control test results (Rotor currents).

Figure 18 shows the *d–q* axis electric currents of the PMSM control system.

According to the test results described above, when faults occur in the electric and position sensors, the faults are influenced by the input voltage and electric current. Similarly, a fault in one part of the control system can influence the other parts.

Figure 18 shows $r_{13}$ and $r_{14}$ from the proposed algorithm during the test.

**Figure 19:**
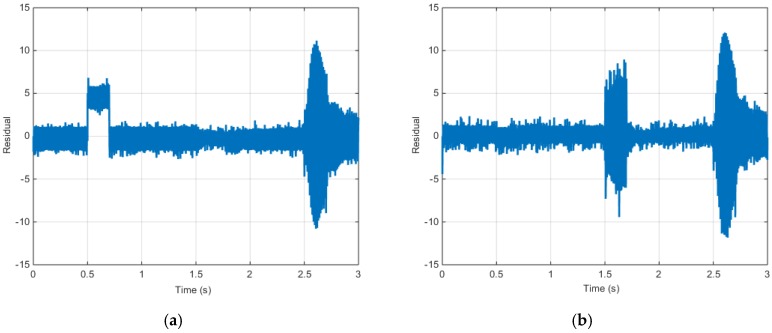
Speed control simulation results (Input voltage): (**a**) Residual ($r_{13}$); (**b**) Residual ($r_{14}$).

When Figure 19 is considered as the fault table listed in Table 3, $r_{13}$ significantly deviates from zero in case of an a-phase electric current sensor fault; meanwhile, $r_{14}$ significantly deviates from zero in case of a b-phase electric current sensor fault. In case of a position sensor fault, both $r_{13}$ and $r_{14}$ significantly deviate from zero. Assuming that only a single fault occurs, residuals $r_{13}$ and $r_{14}$ can be used to detect and separate the faults in the a- and b-phase electric current sensors and the position sensor.

## 4. Integrated Fault-Diagnosis Algorithm

The purpose of the integrated diagnosis is to use the combined high- and low-level fault diagnoses to achieve dual monitoring to specifically classify the fault factors and to distinguish the faults through system performance analysis. The low-level fault diagnosis directly defines the target fault of the individual systems and individually creates residuals to immediately and specifically recognize the fault. However, due to external noise and inaccuracy of the system, it can also trigger a false alarm. To prevent such false alarms, the high-level fault diagnosis can be used to analyze the system control performance while specifically classifying the fault factors using the low-level fault diagnosis.

Using the residual in Equation (19) in the high-level fault diagnosis and the residual in Equations (42) and (43) in the low-level fault diagnosis, the fault-detection flags can be defined as follows:

(44) $$\begin{array}{l} \mathrm{In}\;\mathrm{high}-\mathrm{level}\;\mathrm{fault}\;\mathrm{diagnosis}\\ \begin{array}{cc} if\;\left|r_{1}\right|>th_{1}\;\&\left|r_{9}\right|>th_{9}\;\&\left|r_{i}\right|<th_{i},\;i\neq 1,9 & \mathrm{then}\;f\left(H_{1}\right)=1\\ if\;\left|r_{2}\right|>th_{2}\;\&\left|r_{10}\right|>th_{10}\;\&\left|r_{i}\right|<th_{i},\;i\neq 2,10 & \mathrm{then}\;f\left(H_{2}\right)=1\\ if\;\left|r_{3}\right|>th_{3}\;\&\left|r_{11}\right|>th_{11}\;\&\left|r_{i}\right|<th_{i},\;i\neq 3,11 & \mathrm{then}\;f\left(H_{3}\right)=1\\ if\;\left|r_{4}\right|>th_{4}\;\&\left|r_{12}\right|>th_{12}\;\&\left|r_{i}\right|<th_{i},\;i\neq 4,12 & \mathrm{then}\;f\left(H_{4}\right)=1 \end{array} \end{array}$$

(45) $$\begin{array}{cc} f\;\left|r_{13,1}\right|>th_{ia} & \mathrm{then}\;f(L_{1,1})=1\\ if\;\left|r_{13,2}\right|>th_{ia} & \mathrm{then}\;f(L_{1,2})=1\\ if\;\left|r_{13,3}\right|>th_{ia} & \mathrm{then}\;f(L_{1,3})=1\\ if\;\left|r_{13,4}\right|>th_{ia} & \mathrm{then}\;f(L_{1,4})=1 \end{array}$$

(46) $$\begin{array}{cc} f\;\left|r_{14,1}\right|>th_{ib} & \mathrm{then}\;f(L_{2,1})=1\\ if\;\left|r_{14,2}\right|>th_{ib} & \mathrm{then}\;f(L_{2,2})=1\\ if\;\left|r_{14,3}\right|>th_{ib} & \mathrm{then}\;f(L_{2,3})=1\\ if\;\left|r_{14,4}\right|>th_{ib} & \mathrm{then}\;f(L_{2,4})=1 \end{array}$$

where th refers to the threshold for the fault detection and its value differs according to the type of sensor or actuator. $r_{13,i}$ and $r_{14,i}$ (i=FL, FR, RL, RR=1, 2, 3, 4) refer to the residuals for detecting the electric current and position sensors of the drive motor in each wheel. $f\left(H_{i}\right)$ refers to the fault-detection flag of the high-level fault diagnosis, whereas $f\left(L_{1,i}\right)$ and $f\left(L_{2,i}\right)$ refer to the fault-detection flags of the low-level fault diagnosis. The fault-detection flags become one when the respective residuals exceed the threshold in Equations (44)–(46). Otherwise, they are expressed as zero.

Finally, the integrated fault diagnosis, which is the integrated result of the high- and low-level fault diagnoses of the in-wheel independent drive electric vehicle, is listed in Table 6.

Table 6 lists the final fault diagnosis derived from the simultaneous observations of the fault-diagnosis flags of the high- and low-level fault diagnoses. When the high-level fault diagnosis fault-detection flag $f\left(H_{i}\right)$ is one, it means that the fault is finally diagnosed. Using the corresponding low-level fault diagnosis fault-detection flags $f\left(L_{1,i}\right)$ and $f\left(L_{2,i}\right)$, specific faults can be determined. When $f\left(H_{i}\right)$ is zero and $f\left(L_{1,i}\right)$ or $f\left(L_{2,i}\right)$ is one, because no reduction in the entire system performance occurs, the fault is determined to be tolerable. In other words, the system does not need to be shut down or reconfigured because it can trigger false alarm due to unexpected sensor noise or disturbance to the low-level fault diagnosis. On the other hand, if $f\left(H_{i}\right)$ is one whereas $f\left(L_{1,i}\right)$ and $f\left(L_{2,i}\right)$ are zero, this means that an abnormality occurs from the subject, which is not defined in the low-level fault diagnosis. In this case, we can determine that the risk factor can be recognized by the high level-fault diagnosis. Similarly, the integrated fault-diagnosis method can provide dual monitoring of faults between the high- and low-level fault-diagnosis systems, further enhancing the robustness and stability of the diagnosis procedure. In addition, the integration enables more specific classification of the triggered faults.

## 5. Conclusions

This paper proposed a high-level fault diagnosis of vehicle dynamics and low-level fault diagnosis of the motor system by considering the drive motor of in-wheel independent drive electric vehicles. Simulations were performed to confirm the usefulness of the algorithm. In addition, an integrated fault-diagnosis algorithm, which combines both the above methods, was proposed to prevent false alarm, ensure robustness, and perform more specific classification of faults. Such an integrated fault-diagnosis method cannot only be applied to the drive motor of the in-wheel independent drive electric vehicles but can also be extended to other sub-systems, further enhancing the robustness and stability of the electric vehicle system.

## Figures and Tables

**Table 1:** Fault table of high-level fault diagnosis residual.

	$a_{x}$	$a_{y}$	$\delta_{f}$	$\dot{\psi }$	$v_{x}$	$\omega_{w}$	$T_{mfl}$	$T_{mfr}$	$T_{mrl}$	$T_{mrr}$
$r_{1}$	X	X	X	X	X	X	X			
$r_{2}$	X	X	X	X	X	X		X		
$r_{3}$	X	X	X	X	X	X			X	
$r_{4}$	X	X	X	X	X	X				X

**Table 2:** Fault table of modified high-level fault diagnosis residual.

	$a_{x}$	$a_{y}$	$v_{x}$	$v_{x,gps}$	$\dot{\psi }$	$\omega_{fl}$	$\omega_{fr}$	$\omega_{rl}$	$\omega_{rr}$	$T_{mfl}$	$T_{mfr}$	$T_{mrl}$	$T_{mrr}$
$r_{1}$	X	X	X		X	X	X	X	X	X			
$r_{2}$	X	X	X		X	X	X	X	X		X		
$r_{3}$	X	X	X		X	X	X	X	X			X	
$r_{4}$	X	X	X		X	X	X	X	X				X
$r_{5}$			X		X								
$r_{6}$	X		X		X								
$r_{7}$		X	X		X								
$r_{8}$			X	X									
$r_{9}$	X	X	X		X	X				X			
$r_{10}$	X	X	X		X		X				X		
$r_{11}$	X	X	X		X			X				X	
$r_{12}$	X	X	X		X				X				X

**Table 3:** Fault table of low-level fault diagnosis residual.

	$i_{a}$	$i_{b}$	*θ*
$r_{13}$	X		X
$r_{14}$		X	X

**Table 4:** Interior permanent magnet synchronous motor (IPMSM) model parameter.

Parameter Name	Value (Unit)
Stator resistance (R)	8.296 (mΩ)
*d*-axis stator inductance ($L_{d}$)	0.174 (mH)
*q*-axis stator inductance ($L_{q}$)	0.293 (mH)
Magnet flux linkage ($\phi_{m}$)	71.115 (mV·s)
Inertia (J)	0.089 (kg·m^2^)
Viscous damping (F)	0.005 (Nm·s)
Pole pairs ($n_{p}$)	4 (-)

**Table 5:** PMSM model parameter.

Parameter Name	Value (Unit)
Stator resistance (R)	2/3 (Ω)
*d*-axis stator inductance ($L_{d}$)	0.51/3 (mH)
*q*-axis stator inductance ($L_{q}$)	0.51/3 (mH)
Magnet flux linkage ($\phi_{m}$)	1.25 (mV·s)
Pole pairs ($n_{p}$)	4 (-)

**Table 6:** Fault table of integrated fault diagnosis residual.

Low-Level Fault Diagnosis	High-Level Fault Diagnosis	Fault Isolation	
$f\left(L_{1,1}\right)=1$ and $f\left(L_{2,1}\right)=0$	$f\left(H_{1}\right)=1$	FL Motor	$i_{a}$ Current sensor
$f\left(L_{1,1}\right)=0$ and $f\left(L_{2,1}\right)=1$			$i_{b}$ Current sensor
$f\left(L_{1,1}\right)=1$ and $f\left(L_{2,1}\right)=1$			Position sensor
$f\left(L_{1,2}\right)=1$ and $f\left(L_{2,2}\right)=0$	$f\left(H_{2}\right)=1$	FR Motor	$i_{a}$ Current sensor
$f\left(L_{1,2}\right)=0$ and $f\left(L_{2,2}\right)=1$			$i_{b}$ Current sensor
$f\left(L_{1,2}\right)=1$ and $f\left(L_{2,2}\right)=1$			Position sensor
$f\left(L_{1,3}\right)=1$ and $f\left(L_{2,3}\right)=0$	$f\left(H_{3}\right)=1$	RL Motor	$i_{a}$ Current sensor
$f\left(L_{1,3}\right)=0$ and $f\left(L_{2,3}\right)=1$			$i_{b}$ Current sensor
$f\left(L_{1,3}\right)=1$ and $f\left(L_{2,3}\right)=1$			Position sensor
$f\left(L_{1,4}\right)=1$ and $f\left(L_{2,4}\right)=0$	$f\left(H_{4}\right)=1$	RR Motor	$i_{a}$ Current sensor
$f\left(L_{1,4}\right)=0$ and $f\left(L_{2,4}\right)=1$			$i_{b}$ Current sensor
$f\left(L_{1,4}\right)=1$ and $f\left(L_{2,4}\right)=1$			Position sensor
$f\left(L_{1,i}\right)=0$ and $f\left(L_{2,i}\right)=0$	$f\left(H_{i}\right)=1$	Intolerable and not isolatable	
$f\left(L_{1,i}\right)=1$ or $f\left(L_{2,i}\right)=1$	$f\left(H_{i}\right)=0$	Tolerable and isolatable	
